# Investigation of internal damage evolution in gneiss considering water softening

**DOI:** 10.1038/s41598-023-39664-8

**Published:** 2023-08-04

**Authors:** Changhai Sun, Bingxin Xie, Rui Wang, Xianghui Deng, Jin Wu

**Affiliations:** 1 Shaanxi Transportation Holding Group Co., Ltd., Xi’an, 710065 China; 2 School of Civil and Architecture Engineering, Xi’an Technological University, Xi’an, 710021 China

**Keywords:** Civil engineering, Materials science

## Abstract

In soft rock tunnels, there are often large deformations during construction, especially when the groundwater seepage and softens the surrounding rock. For achieving the purpose of studying the softening effect of water immersion on strength and stability of surrounding rock, 15 rock samples were selected for physical and mechanical tests under 5 conditions: natural state and free immersion for 1, 3, 6, and 9 months, and nuclear magnetic resonance technology(NMR) was also adopted to test the internal pore structure of specimens with different immersion durations, thus the micro structure features of the gneiss, such as the NMR relaxation time T2 spectrum distribution, porosity, and pore volume ratio of different pore sizes under water softening were then obtained. The NMR results shows that the longer the free immersion duration of the rock sample, the greater the porosity; at the same time, the number of micropores in the rock gradually decreases under the interaction of water and rock, and the mesopores increase slightly first and then decrease all the time. The number of macropores is gradually increasing. When the immersion duration is 6 months, the number of macropores begins to increase significantly, and the mechanical properties of the specimens begin to drop significantly. By 9 months, the proportion of macropores in the rock has reached 57.6%. The results showed that the number growth of macropores is the root cause of the macroscopic failure of rock sample. The study results have significance for on-site construction in water-rich areas.

## Introduction

Under the special natural conditions in water-rich areas, geotechnical engineering construction will be inevitably affected by groundwater. Compared with other rocks, soft rock has the characteristics of broken structure and high expansibility, and the long-time immersion in water will cause self-bearing capacity decrease and obvious strength decrease in terms of the mechanical properties^1^, while the internal damage is manifested by the continuous change of complex pore structure existed in the rock during water softening process, which determines mechanical properties and fracture characteristics of the macroscopic in rock^2^. Therefore, if the internal damage characteristics of soft rock under immersion softening can be accurately grasped, it will be of great significance to analyze soft rock deformation and failure.

Currently, many domestic and foreign scholars have conducted a lot of experiments and theoretical studies on the impact of softening effect by immersion in water on properties and internal damages of rock. Regarding the impact on mechanical properties, Hashiba et al. carried out compression and tensile tests to r investigate whether the deformation and failure of mountain andesite can be influenced by water saturation. The results shows that the compressive strength and tensile strength of rocks decrease linearly with the logarithm of water saturation^3^. Hasan et al. used the saturated caliper method to conduct water–rock interaction experiments and explored how water affects the strength mechanism of granite under different grades of weathering. The results show that when water exists in the rock microstructure, wetting and drying processes will occur. In this process, lots of micropores are generated, resulting in the weakening of the rock structure and strength^4^. To study how the physical and mechanical properties of sandstone change under the influence of water, Zhou et al. used uniaxial compression tests to study sandstones with different moisture content. It shows that the uniaxial compressive strength and elastic modulus of sandstone are inversely proportional to the moisture content, which means the presence of water will soften the rock and weaken mechanical properties^5^. To find out the whole process and mechanism of the weathering and destruction of Longyou Grottoes by the change of water environment, Shao et al. conducted mechanical properties and elastic wave tests of sandstone under different water-bearing conditions. The research results show that with moisture content increasing, the strain softening characteristics weakened, while the peak strength and the modulus of elasticity decreased exponentially^6^. Considering that the presence of water will cause a gradient change in the internal temperature of the rock, which will further affect its mechanical property, Yu et al. carried out multiple cooling and heating cycles on granite, and conducting static mechanical experiments, the test shows that with the test is carried out in multiple cycles under cold and hot conditions, the strength of the granite decreases significantly^7^. Rabat et al. evaluated the effect of water on the peak and residual compressive strengths and tangent tensile modulus of materials at different confining pressures by triaxial compression tests, and found that under different confining pressure conditions, water would cause the strength and tensile modulus of the tested rock sample to decrease significantly^8^. Verstrynge et al. researched the impact of moisture on the damage form of ferrous sandstone from both the microscopic and macroscopic aspects, and the experimental analysis was carried out on different levels. It was found that the strength and stiffness of iron sandstone of lower quality decreased more obviously^9^. In the acoustic emission control creep test, the adsorption of water by the sandstone changed the behavior of the specimen from metastable creep failure to accelerated failure. To explore the mechanism of action of water on the strength of olivine and internal structural changes. Tielke et al. conducted two deformation tests on olivine was subjected under anhydrous and hydrated conditions, and found that the crystal would be hydrolyzed and weakened, and the strength significantly reduced^10^.

There are also many studies about the internal damage of rock by softening effect of immersion in water. Li et al. carried out real-time observations throughout the process to investigate the microscopic damage of the gypsum breccia under uniaxial compression load, and the results show that the micro-cracks of initial damage caused by the influence of water environment is an important factor in the softening effect of water on the mechanical properties of swelling soft rocks^11^. Nara et al. used load relaxation method to study the crack growth rate under constant temperature and humidity conditions. It was found that, when under a given stress intensity factor, the relative humidity at a constant temperature increased by three or four times, the crack growth rate increases exponentially^12^. Quan et al. used CT to obtain the whole process of real-time interaction between mudstone and water. The results show that discontinuous nature of creating tiny pores provides the original path for water intrusion into the rock. The natural water then causes the volume expansion of the clay minerals and the dissolution of carbonates, which affects the expansion and connection of the driving cracks, and ultimately leads to the destruction of the mudstone^13^. On the basis of nuclear magnetic resonance (NMR), Sun et al. analyzed the internal pore changes and deformation mechanisms of schist under the influence of immersion durations and the concentration of the immersion solution. The results show that under the interaction between water and rock, the distribution of internal pores in the rock further affects the stability of the slope, which should be taken seriously^14^. Xing et al. injected water pressure into the rock surface with pre-existing defects, and observed the distribution range and shape size of cracks by CT and SEM techniques. After analysis, it is found that the water pressure will accelerate the further expansion of the original crack, and the main form of force is expressed as tension^15^. Researching on the change of rock strength based on different immersion durations, Zhu et al. took gypsum rock as the experimental rock sample, performed conventional compression and electronic microscopic tests on rock samples under different immersion durations. The results show that, as the immersion duration increases, the bonds in the microporous fractures and the crystals at the tips of the microcracks will weaken due to hydrolysis, and the microcracks will continue to expand^16^.

In summary, the studies mentioned above mainly concentrated in the softening failure form of rock or the change in mechanical properties caused by immersion. There is no in-depth analysis on the internal damage of rock, and the distribution and proportion of different pores in rock are the root cause that often leads to softening until macroscopic damage. However, there are still few studies about this.

Therefore, on the basis of the theory of rock damage, this paper takes gneiss as the research object, carries out indoor uniaxial compression tests and NMR tests, and analyzes the uniaxial compressive strength, elastic modulus, T2 spectrum, porosity of the rock, and the change in the proportion of pore volume of different apertures at different immersion durations. And combined with the equivalent strain hypothesis, it defines the damage variable on the basis of elastic modulus, fits the relationship between immersion durations and damage variable, analyzes the characteristics of pore size distribution and the damage and deterioration mechanism of pore water on pore structure of gneiss under softening effect of waters, and the degree of influence and damage characteristics of different sizes of pores on the internal damage of rocks are revealed.

## Experimental program

### Specimen preparation and numbering

The selected rock sample is gneiss, taken from a railway tunnel from Zhenjiangguan to Songpan in Sichuan Province, with good uniformity and integrity, ranging from light gray to dark gray. The main mineral components are quartz, feldspar, chlorite, sericite, etc. The rock is relatively soft, fragile by hammering, and the core is nubby. Among these mineral compositions, feldspar is the composite mineral mainly composed of magnesium (0.32%), potassium (0.67%), calcium (0.18%) and other elements. It is easy to dissolve under immersion. Therefore, in the immersion process, water–rock interaction will occur. According to conventional rock mechanics test standards, 15 pieces and 5 groups of 100 mm × 50 mm cylindrical specimens were processed according to the height-diameter ratio of 2:1. The specimen number is S-x-y, in which x represents the free immersion duration of the test block, and the value is 0, 1, 3, 6, 9, y represents the ordinal number of the test block, the value is 1, 2, and 3. Select the numbered test block for the uniaxial compression deformation test of rock sample. The other part is processed into 3 pieces of 3 groups of 50 mm × 50 mm cylindrical samples according to the height-diameter ratio of 1:1. The specimen number is S-i, in which i represents the ordinal number of the test block, and the value is 1, 2, 3. Select the numbered test block for NMR test.

### Testing method and program

The 15 rock samples taken on site were used under natural state and after free immersion for 1, 3, 6, and 9 months, respectively, using the YA-2000 digital display pressure testing machine to complete the uniaxial compression test. The remaining three rock samples were tested with MesoMR23-060H-I medium-size NMR imaging analyzer with a resonance frequency of 23.415 MHz, a coil diameter of 70 mm, and a magnet temperature of 32 °C under the same five conditions indicated previously. It should be noted that after each measurement period, a rock sample needs to be taken out, and the surface water is wiped off before the nuclear magnetic resonance test is performed to obtain the changes in porosity, lateral relaxation time T2 spectrum before and after immersion.

## Analysis of test results

### Analysis of uniaxial compression deformation test results

**Figure 1:**
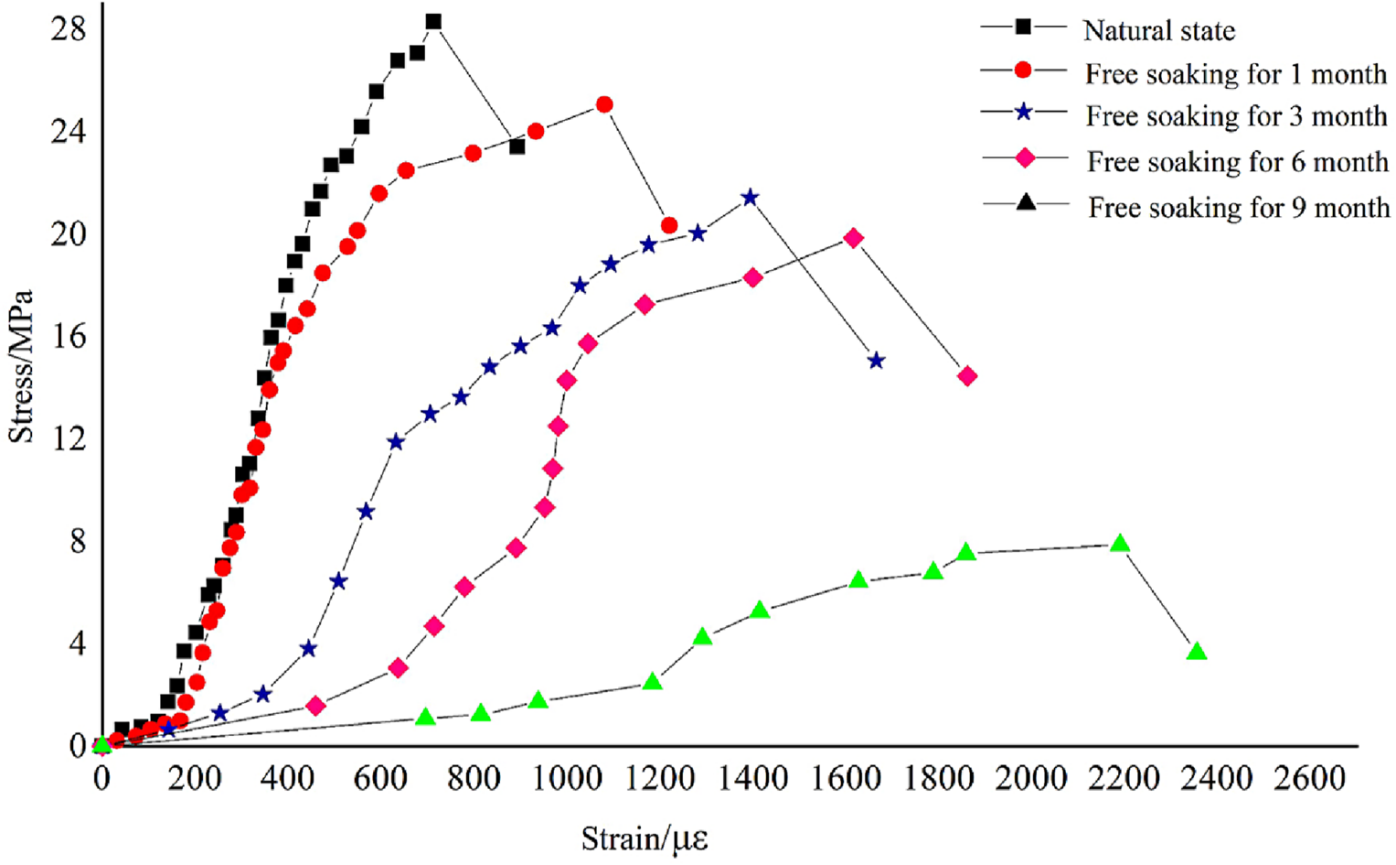
Uniaxial compressive stress–strain curves of rock samples under different free immersion durations.

The data obtained from each group of uniaxial compression tests were processed and plotted as stress–strain curves, which is shown in Fig. 1 (in order to comparative analysis, a typical curve was selected as a representative curve for each group).

Through uniaxial compression deformation tests of rock samples within different free immersion durations, how the mechanical properties of rock samples change under the influence of softening effects caused by immersion is investigated, the rock damage is analyzed from a macro perspective as well. The test results are mainly analyzed from the changes in mechanical properties. By studying the elastic modulus E, the internal damage of rock is quantitatively analyzed.

To make the constitutive equation of the rock sample uncomplicated, considering the damage factor, based on the equivalent strain hypothesis proposed by the French scholar Lemaitre, which means the full stress is replaced by the effective stress, the strain and full stress of the obtained non-destructive material act on the strain produced by the lossy material is equivalent^17^. This hypothesis has been widely used to study the deformation and damage characteristics of rock, concrete and other materials^18,19^.After deduction, the damage variable expression relied on the elastic modulus is obtained:

1 $$D = 1 - E^{\prime } {/}E$$

where E means the elastic modulus of the non-destructive material, E` means the elastic modulus of the damaged material, D means the damage variable, and 0 ≤ D ≤ 1.

Although the application of this damage variable related to macro-mechanical behavior is more convenient, it is limited for many materials have initial damage in actual engineering practices. Taking the rock sample in this article as an example, it is the material with natural damage. There is hardly any damage-free rock, so for natural damage materials like rocks, it is more difficult to measure the elastic modulus of the non-destructive material in the above formula. Therefore it needs to be noted that the elastic modulus E in the above calculation formula is totally different with the elastic modulus in traditional damage constitutive equation: the traditional damage constitutive equation takes the elastic modulus of a truly compact and undamaged rock, which is more difficult to be measured in practice, and the damage constitutive equation given here is the elastic modulus in natural state of rock (baseline damage or initial damage), which is relatively easy to be measured.

Based on the field test data, the rock damage variables of each flooding stage are calculated by formula (1), and the results are shown in Table 1.

**Table 1:** Mechanical indexes and damage variables of rock samples within different free immersion durations.

Free soaking time/month	Serial number	Uniaxial ultimate compressive strength/MPa	Average value of uniaxial ultimate compressive strength/MPa	Elastic modulus E/GPa	Elastic modulus E initial value/GPa	Damaged material elastic modulus E′/GPa	Damage variable D
0	S-0-1	30.81	28.187	102.172	103.362	103.362	0
	S-0-2	25.46		98.165			
	S-0-3	28.29		109.749			
1	S-1-1	28.51	26.823	89.648	103.362	88.156	0.146
	S-1-2	26.91		87.830			
	S-1-3	25.05		86.991			
3	S-3-1	23.78	23.137	63.622	103.362	63.524	0.385
	S-3-2	24.21		62.262			
	S-3-3	21.42		64.689			
6	S-6-1	18.83	18.531	40.954	103.362	39.857	0.653
	S-6-2	19.85		38.003			
	S-6-3	17.01		40.614			
9	S-9-1	5.86	6.300	8.935	103.362	9.105	0.912
	S-9-2	7.64		11.490			
	S-9-3	5.50		6.889			

Table 1 shows that the uniaxial ultimate compressive strength and modulus of elasticity of the rock samples are affected to different degrees with the increase of free immersion duration, and both of them showed a decreasing trend. When the free immersion is for 9 months, the elastic modulus of rock samples is only 9.105 GPa, a decrease of 91.2% compared to that under the natural state, the uniaxial compressive strength is only 6.3 MPa, a decrease of 77.7% compared to that under the natural state, and the damage variable D is 0.912 at this point. This indicates that the internal damage is serious and the rock almost loses its original strength. The compressive strength curves obtained from uniaxial tests can also be used as evidence.

The uniaxial compression deformation damage of rock samples under different free immersion durations all exhibit brittle weak face splitting damage, which is flaky after damage. This form of damage occurs mainly due to the thin lamellar structure of the rock sample itself and the orientation of its internal joints parallel to the section of the specimen. The damage form of this rock sample is not related to its softening time by immersion, but the degree of damage is increasing with the increase of immersion time. With the prolongation of free water immersion duration, the uniaxial compression damage appears more and more cracks, and the degree of damage becomes bigger and bigger, even some of the specimens are flaky or "compression rod" extrusion after compression damage. The shear fracture surface is also accompanied by gray powder formed by strong friction, which indicates that the number of internal pores is increasing and the pore size is increasing after the free immersion of the rock sample with the extension of the free immersion time. The weak structural surface and the joint surface are softening continuously, the cementing force and mechanical bite force are gradually decreasing, the local stress concentration phenomenon is obvious, and The degree of damage increases gradually..

**Figure2:**
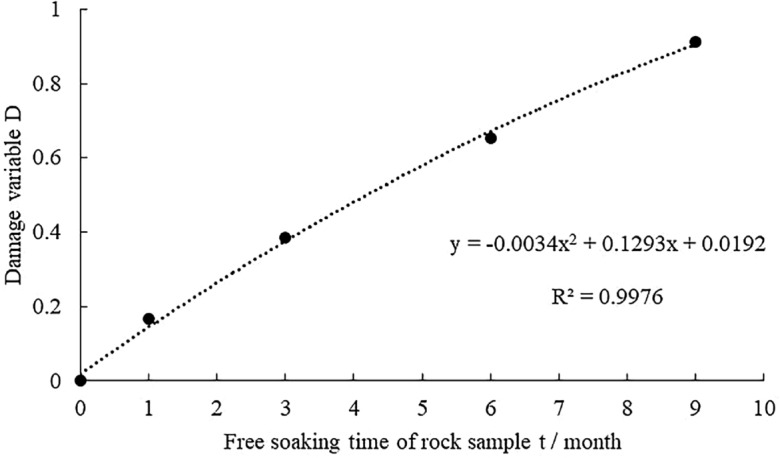
Fitting curve diagram of the damage variable of rock sample and different free immersion durations.

Data on the damage variable of the rock sample within different free immersion durations shown in Table 1, the damage variable and the corresponding free immersion durations are fitted, which is shown in Fig. 2.

Figure 2 indicates that the fitting of the damage variable of rock sample and free immersion durations is basically consistent, the fitting correlation coefficient R2 = 0.9976, and the fitting relationship is a quadratic polynomial, shown as formula (2).

2 $$D = - 0.0034t^{2} + 0.1293t + 0.0192$$

The errors of the fitted predicted values of the damage variables of the rock samples under different free immersion durations and the measured values of the tests were compared and analyzed, as shown in Table 2. From Table 2, it can be seen that the fitted and measured values are within a reasonable range, and thus the reliability of Eq. 2 can be verified.

**Table 2:** Error analysis of damage variables for rock samples at different free immersion durations.

Free immersion durations/months	Number	Measured value	Predicted value	Error rate/%
1	S-1-1	0.132	0.145	8.76
	S-1-2	0.149		2.68
	S-1-3	0.158		8.23
3	S-3-1	0.384	0.377	1.83
	S-3-2	0.398		5.28
	S-3-3	0.374		0.80
6	S-6-1	0.604	0.673	10.25
	S-6-2	0.632		6.49
	S-6-3	0.607		9.81
9	S-9-1	0.914	0.908	0.66
	S-9--2	0.889		2.13
	S-9-3	0.933		2.68

The damage variable of rock sample showed a gradual increasing trend with the extension of free immersion durations, and in the 9th month, D was close to 1, and the rock was approaching the failure state.

### Analysis of NMR test results

The time for a proton to move in the presence of a magnetic field is called the relaxation time, which is represented by T2. T2 relaxation time is not only related to the characteristics of the atomic nucleus itself, but also affected by the pore volume and surface area. Generally speaking, relaxation time decreases as the pore size decreases, so the observed T2 spectrum distribution can represent the pore size distribution of rock sample after a reasonable scale or conversion. The area under the curve can represent the percentage of the pore volume.

Since free relaxation, surface relaxation and diffusive relaxation are the three relaxation mechanisms of rock pore fluids, T2 can be represented as:

3 $$\frac{1}{{T_{2} }} = \frac{1}{{T_{2free} }} + \frac{1}{{T_{2sur} }} + \frac{1}{{T_{2diff} }}$$

Through the research of many scholars, it is found that free relaxation and diffusion relaxation are very small compared with surface relaxation. Therefore, T2 relaxation is mainly determined by surface relaxation. Therefore, Eq. (3) is able to be simplified as:

4 $$\frac{1}{{T_{2} }} = \frac{1}{{T_{2sur} }} \approx \rho_{2} \left( \frac{S}{V} \right)_{pore}$$

where V means the pore volume (cm^3^); S means the pore surface area (cm^2^); ρ_2_ means the lateral surface relaxation strength (μm/ms).

According to formula (4), the aperture can be calculated. At the same time, it can be seen from formula (4) that there is a certain proportional relationship between the lateral relaxation time rate 1/T2 and the pore surface area and volume of measured material. Therefore, through the distribution of transverse relaxation time T2 inside the test material, the pore size and distribution information inside the test material can be indirectly calculated. The results are mainly analyzed from the following two aspects: the change of porosity of the rock sample within different free immersion duration sand the change of pore distribution of the rock sample within different free immersion durations.

1. Analysis of porosity change

**Figure 3:**
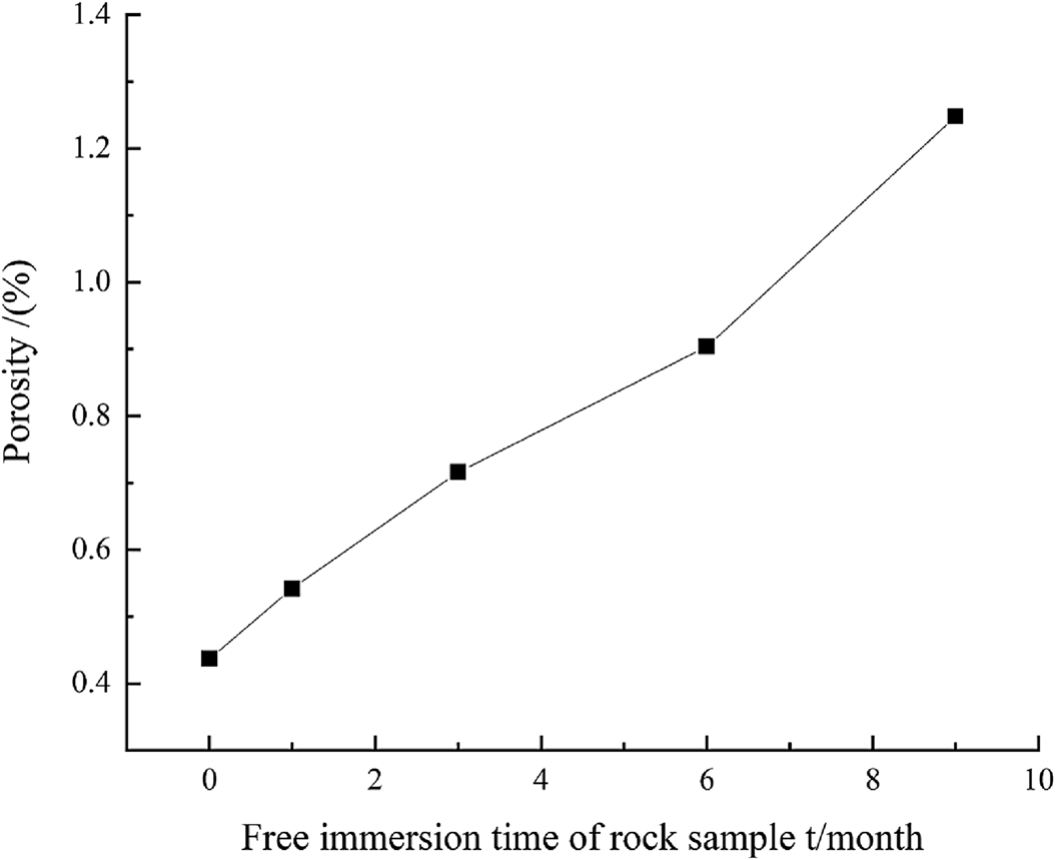
Mean porosity variation curves of rock samples under different free immersion durations.

The internal porosity of rock sample within different free immersion durations obtained from the NMR test results are shown in Table 3 and Fig. 3.

**Table 3:** Porosity of rock samples within different free immersion durations.

Serial number	Porosity in natural state/%	Average porosity/%	Free water porosity/%							
			One month	Average value	Three months	Average value	Six months	Average value	Nine months	Average value
S-1	0.524	0.437	0.607	0.542	0.718	0.716	0.853	0.904	1.165	1.248
S-2	0.452		0.552		0.756		0.896		1.146	
S-3	0.336		0.467		0.673		0.964		1.432

Table 3 and Fig. 3 show that the porosity changes of rock sample with the extension of free immersion durations show the following two characteristics:

1. The porosity of the rock samples is positively correlated with the free immersion duration. The porosity of rock sample in the natural state is about 0.437%, and those under the conditions of free immersion for 1 month, 3 months, 6 months and 9 months are about 0.542%, 0.716%, 0.904% and 1.248% respectively, which show the increases of 0.105%, 0.279%, 0.467% and 0.811% respectively compared with the porosity in the natural state.
2. When the free immersion duration reaches 6 months, the porosity growth rate of the rock samples starts to increase. During the 1st month of free immersion, the porosity has a small change, which increased by 24.0% compared to the natural state. In the 9th month, the porosity has increased by 185.6% compared to that in the natural state and by 38.1% compared to that in the 6th month. The porosity of rock sample has changed significantly before and after, and the internal damage is serious.

2. Analysis of pore distribution changes

Because the proportion of different pore sizes in rock will continue to change with the time extension of free immersion. The proportion of macropores has a greater impact on rock mechanical properties than micropores and mesopores. And the difference in the size, shape as well as distribution of the pores inside the rock will also cause differences in the distribution of pore water, which will in turn cause different forms of pore structure damage and deterioration. Therefore, the well understanding to characteristics of rock pore size distribution is significant to analyzing the internal damage law of rock under water softening effect (Supplementary Material).

**Figure 4:**
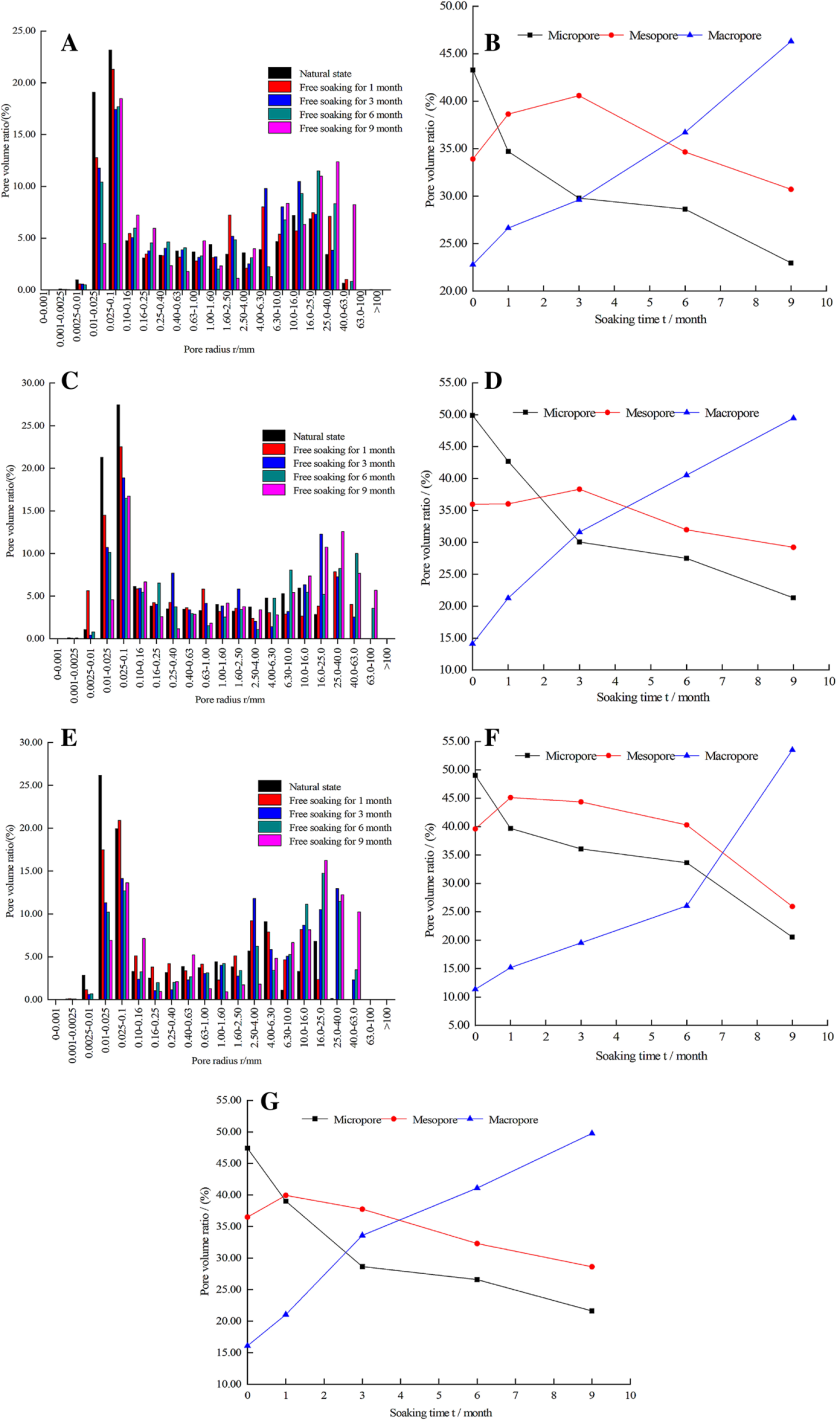
Distribution and change of pore volume ratio of rock samples within different free immersion durations. (**A**) S-1. (**B**) S-1 Changes in the volume ratio of each pore. (**C**) S-2. (**D**) S-2 Changes in the volume ratio of each pore. (**E**) S-3. (**F**) S-3 Changes in the volume ratio of each pore. (**G**) Change in the average proportion of each pore volume.

Referring to the laboratory capillary pressure measurement pore radius classification method and combining with the pore size distribution characteristics of the gneiss used, this paper divides the gneiss pore size into three intervals for statistical purposes: the r of micropores is less than 0.01 µm; the r of mesopores is between 0.01 µm and 0.05 µm; and the r of macropores is between 0.05 µm and 1 µm^20,21^. According to formula (4) and the T2 spectrum of rock sample within different free immersion durations measured by nuclear magnetic resonance, the volume distribution of different pore radius is then obtained. Analyzed the results and plotted the histogram of pore radius and pore volume percentage of diagenetic samples, and the broken line chart of free immersion durations and pore volume percentage are shown in Fig. 4.

Figure 4 indicates that the distribution of pore radius and the proportion of pore volume of micropores, mesopores and macropores in the rock sample within different free immersion durations show the following two changes:

1. The pore radius distribution inside the rock sample is between 0 μm and 100 μm, and most of the pore radius is in the range of 0.01 μm ~ 40 μm.
2. In natural state and under free immersion conditions for 1, 3, 6, and 9 months, the volume of micropores in rock samples accounted for 47.41%, 39.03%, 31.98%, 29.92%, and 21.61% respectively, that of mesopores 36.49%, 39.93%, 41.10%, 35.64%, and 28.63% respectively, and macropores 16.10%, 21.04%, 26.92%, 34.43%, and 49.76% respectively. It shows that, with the extension of free immersion duration, the number of micropores gradually decreases, mesopores firstly increases and then decreases, and macropores gradually increases. This is because the particle size and mineral morphology inside the rock will affect its pore characteristics. With the extension of soaking time, many mineral components inside the phyllite, such as feldspar, are easy to dissolve under the soaking of water. The particles and cements filled in the pores are easy to fall off and migrate under the action of fluid shear force, resulting in the original small pores inside the rock. The pores are interconnected and eventually become medium pores and large pores, and the degree of damage gradually increases.When the immersion duration reaches 6 months, the number of macropores begins to increase significantly, and the mechanical properties of the specimens begin to decrease significantly. By 9 months, the proportion of macropores in the rock has reached 57.6%, which indicates that the micropores in rock have, under the impact of water–rock reactions, gradually turned into mesopores and macropores, and the increase of macropores will aggravate the internal damage of rock and eventually lead to its macroscopic destruction.

## Conclusion

In this paper, based on rock damage and considering the softening effect of immersion, common macro-mechanical tests and micro-NMR tests have been carried out on gneiss in natural state and under free immersion conditions for 1, 3, 6, and 9 months. Combining with the macroscopic and microscopic test results, the relationship between the internal damage of rock and the free immersion durations is analyzed and the rule is summarized. The following conclusions were obtained:

1. As the free immersion duration increases, the elasticity modulus and uniaxial compressive strength of rocks showed a decreasing trend Starting from the 6th month of free immersion, the uniaxial compressive strength and elastic modulus of decrease sharply with a rate of 66.0% and 77.2% respectively. By the 9th month, the elastic modulus is only 9.105 GPa, which is a 91.2% decrease compared with that in the natural state, and the uniaxial compressive strength is only 6.3 MPa, which is a 77.7% decrease compared with that in the natural state. The test data shows that water influences the mechanical properties and internal damage of rock greatly.
2. Combining with the related principles of damage mechanics and based on Lemaitre's equivalent strain assumption, the continuous elastic modulus E is used to quantitatively describe the damage to gneiss caused by immersion durations, and fit the relationship between the damage variable D and the immersion durations t expression.
3. From the NMR test results of gneiss under the different immersion durations, the porosity of the rock sample in its natural state and under free immersion conditions for 1, 3, 6, and 9 months are 0.437%, 0.542%, 0.716%, 0.904%, and 1.248%, respectively. The porosity of the rock is positively correlated with the immersion duration. Besides, when the immersion duration reached a certain critical value, the porosity growth rate of the rock samples began to show a significant increase, and the porosity change was the largest from 6 months of immersion duration, and the porosity growth rate of the rock samples was 38.1% from 6 months of immersion to 9 months of immersion.
4. The proportion of pores in rock sample is constantly changing with the increase of immersion duration. Micropores number is gradually decreasing, medium pores firstly increases and then decreases, and macropores gradually increases. By the 6th month, the number of macropores begins to increase significantly, and the mechanical properties of the specimens begins to decline significantly. By the 9th month, the proportion of macropores in the rock has reached 57.6%, because that, with hydration inside the rock, the original micropores will gradually turn into mesopores and macropores. For the proportion of macropores is decisive for the mechanical properties and internal damage of rocks, the degree of damage inside the rock can be quantitatively described by the proportion of macropores.

### Supplementary Information

Supplementary Information.

## Data Availability

All data generated or analysed during this study are included in this published article [and its supplementary information files].
