# Supplementary material for: Investigation of internal damage evolution in gneiss considering water softening
Source: Sci Rep. 2023 Aug 4;13:12672. doi: 10.1038/s41598-023-39664-8 (PMC10403494; doi:10.1038/s41598-023-39664-8)
Supplement: Supplementary file 1 — Supplementary Information. [file 41598_2023_39664_MOESM1_ESM.pdf]

This file contains raw data of Nuclear magnetic resonance test and mechanical indexes of rock samples under different free soaking durations.

Data list:

Table A is raw data of mechanical indexes of rock samples under different free soaking durations.In the Manuscript,it shows the Figure 2 and Figure 3.

Table B -Table D is the raw data of Nuclear magnetic resonance test.In the Manuscript,Table B shows the Figure 5(A) and Figure 5(B) ; Table C shows the Figure 5(C) and Figure 5(D);Table D shows the Figure 5(E) and Figure 5(F).

Table E is the raw data of the water content of the rock sample with a diameter of 50 mm and a height of 100 mm in the natural state and after soaking.

Figure F(a) and (b) shows the test specimens used for the tests. Figure 1(a) shows the standard specimens made for the determination of mechanical properties such as uniaxial compression of rock samples according to the conventional rock mechanical test standards. Figure 1(b) shows the test specimens for the determination of NMR

Figure G(a)-Figure G(d) shows the uniaxial compression failure modes of rock samples under different free soaking durations.

Free soaking time/month	Serial number	diameter /mm	height /mm	volume /cm^3	Immersion quality/g	Uniaxial ultimate compressive strength/MPa	Average value of uniaxial ultimate compressive strength/MPa	Peak strain/με		Elastic modulus E/GPa	Elastic modulus E initial value/GPa	Damaged material Elastic modulus E`/GPa	Damage variable D
								Transverse peak strain	Longitudinal peak strain				
	S-0-1	50.26	99.88	198.06	543.59	30.81		228.5	617	102.172			
0	S-0-2	50.2	100.5	198.89	537.75	25.46	28.187	307	830	98.165	103.362	103.362	0
	S-0-3	50.2	99.88	197.59	544.06	28.29		263	712.5	109.749			
	S-1-1	50.24	100.1	198.36	546.98	28.51		376	896	89.648			
1	S-1-2	50.2	100.1	198.04	547.18	26.91	26.823	387	923.5	81.783	103.362	86.141	0.167
	S-1-3	49.86	100.2	195.5	559.33	25.05		462	1081	86.991			
	S-3-1	50.24	100.3	198.65	558.78	23.78		532	1268.5	63.622			
3	S-3-2	50.26	100.3	198.89	552.45	24.21	23.137	498	1186	62.262	103.362	63.524	0.385
	S-3-3	50.24	100.2	198.55	563.95	21.42		585	1394	64.689			
	S-6-1	50.26	100.2	198.75	546.94	18.83		768	1812	40.954			
6	S-6-2	50.2	99.22	196.28	548.45	19.85	18.531	712	1617	38.003	103.362	39.857	0.653
	S-6-3	50.24	100.9	199.92	563.9	17.01		772	1536	40.614			
	S-9-1	49.88	98.37	192.13	546.86	5.86		986	2190.5	8.935			
9	S-9-2	49.86	99.24	193.67	541.88	7.64	6.300	946	1916	11.49	103.362	9.105	0.912
	S-9-3	50.24	100.8	199.74	545.06	5.5		1102	2268	6.889			

Table A Mechanical indexes of rock samples within different free immersion durations
(Raw data of Figure 2 and Figure 3)

Aperture/μm	Natural state	Free Soaking for 1 month	Free Soaking for 3 month	Free Soaking for 6 month	Free Soaking for 9 month
0-0.001	0.000266456	0.000158497	0.000152376	0.000133801	0
0.001-0.0025	0.092503532	0.054985024	0.052954778	0.046484856	0
0.0025-0.01	0.972577717	0.581221195	0.562519581	0.490426127	0
0.01-0.025	19.06797951	12.7705193	11.75862482	10.40921862	4.498455895
0.025-0.1	23.15319141	21.29666559	17.42607233	17.68422878	18.4721949
0.10-0.16	4.761707765	5.445950637	5.050952191	5.956168595	7.218297945
0.16-0.25	3.088696996	3.462371759	3.782571958	4.542905291	5.938996188
0.25-0.40	3.333326355	3.29821972	4.040457487	4.627095129	2.348451259
0.40-0.63	3.765983651	3.169388925	3.865047423	4.064259864	1.776393011
0.63-1.00	3.66614338	2.793692992	3.149751953	3.287886128	4.73265312
1.00-1.60	4.381714023	3.127325567	3.207157887	2.008423693	2.317797679
1.60-2.50	3.448261316	7.233197032	5.178644447	4.829548761	1.128218736
2.50-4.00	3.577985042	2.104648184	2.5229575	3.112497757	3.970518579
4.00-6.30	3.890977943	8.011038569	9.786734162	2.222633469	1.287241881
6.30-10.0	4.661441914	5.377635032	8.006499129	6.749693244	8.350733497
10.0-16.0	7.195654994	5.70395981	10.46995258	9.300185175	6.328709367
16.0-25.0	6.867753206	7.450105379	7.306917948	11.51027036	10.98925298
25.0-40.0	3.420962545	7.098836345	3.832031456	8.321546382	12.36230898
40.0-63.0	0.652872252	1.020080438	0	0.836393966	8.236691865
63.0-100	0	0	0	0	0.043084115
>100	0	0	0	0	0
Micropore	43.28651862	34.70354961	29.80032388	28.63049219	22.9706508
Mesopore	33.91479647	38.64583339	40.58427501	34.65141869	30.7185684
Macropore	22.79868491	26.650617	29.61540111	36.71808912	46.31078081

Table B S-1 Nuclear magnetic resonance test raw data
(Raw data of Figure 5(A) and Figure 5(B))

Aperture/μm	Natural state	Free Soaking for 1 month	Free Soaking for 3 month	Free Soaking for 6 month	Free Soaking for 9 month
0-0.001	0.000293621	0.000172128	0.000114536	0.000218419	0
0.001-0.0025	0.101757724	0.059820821	0.039948491	0.075767842	0
0.0025-0.01	1.066206936	5.636843872	0.436528713	0.803498837	0
0.01-0.025	21.30088085	14.47795748	10.71665219	10.1376777	4.574645476
0.025-0.1	27.43649229	22.52499636	18.86011192	16.47742476	16.73235584
0.10-0.16	6.146799948	5.847882887	5.914276353	5.454822035	6.659177686
0.16-0.25	3.820256275	4.240357098	4.042463383	6.534196005	2.587985837
0.25-0.40	3.499821724	4.254760851	7.693838682	3.752848434	1.172664376
0.40-0.63	3.464688377	3.634367154	3.394376372	2.948260206	2.87556413
0.63-1.00	3.317927327	5.828026059	4.153253888	1.511913053	1.821012608
1.00-1.60	4.013775498	3.204074217	3.84800446	2.548858367	4.166255405
1.60-2.50	3.208560458	3.564589677	5.833262835	3.42084845	3.760722232
2.50-4.00	3.721605835	2.393685938	2.036963709	1.056654172	3.384532525
4.00-6.30	4.778498977	3.06152481	1.418164448	4.746748163	2.804778613
6.30-10.0	5.280826749	2.897782627	3.203485036	8.064768149	5.420298823
10.0-16.0	5.95203254	2.665795945	6.325130064	5.465495409	7.372408701
16.0-25.0	2.845294104	3.817574237	12.26298935	5.2	10.74076546
25.0-40.0	0.044280773	7.859352265	7.280317344	8.24	12.57505802
40.0-63.0	0	4.030435574	2.54011823	10	7.67177427
63.0-100	0	0	0	3.56	5.68
>100	0	0	0	0	0
Micropore	49.90563141	42.69979066	30.05335585	27.49458756	21.30700132
Mesopore	35.97193442	36.02926869	38.33460413	31.97514889	29.23269341
Macropore	14.12243417	21.27094065	31.61204002	40.53026356	49.46030527

Table C S-2 Nuclear magnetic resonance test raw data
(Raw data of Figure 5(C) and Figure 5(D))

Aperture/μm	Natural state	Free Soaking for 1 month	Free Soaking for 3 month	Free Soaking for 6 month	Free Soaking for 9 month
0-0.001	0.000230976	0.000317438	0.000169319	0.000189269	0
0.001-0.0025	0.080083338	0.110196988	0.05860451	0.065383805	0
0.0025-0.01	2.833784337	1.160375093	0.605223127	0.671005712	0
0.01-0.025	26.17462519	17.50121142	11.29707624	10.21421799	6.91545478
0.025-0.1	19.93549655	20.91545741	14.12730104	12.69527115	13.63628637
0.10-0.16	3.290029592	5.113862118	2.385332519	3.244908368	7.124207182
0.16-0.25	2.51579997	3.801726102	1.056430483	1.980181539	0.94734881
0.25-0.40	3.167167646	4.193985979	1.166256303	2.020041957	2.105518451
0.40-0.63	3.863970577	3.375292873	2.306691634	2.654797149	5.211502924
0.63-1.00	3.738410469	4.144128801	3.066294363	3.113032093	1.283958585
1.00-1.60	4.424276391	2.289266292	3.997205367	4.207904614	0.910431436
1.60-2.50	3.835711212	5.110261795	2.755016563	3.409662723	1.727455939
2.50-4.00	5.661902437	9.209016727	11.78764665	6.245572711	1.809780475
4.00-6.30	9.104860474	7.889022193	5.846647329	3.425894186	4.817589413
6.30-10.0	1.113657818	4.654214958	5.066278853	5.257684401	6.650573268
10.0-16.0	3.308075561	8.166663816	8.687612126	11.10876861	8.161909935
16.0-25.0	6.81341427	2.365	10.5113192	14.74397362	16.2378279
25.0-40.0	0.138503193	0	12.95853474	11.44939674	12.22880454
40.0-63.0	0	0	2.320359637	3.492113373	10.23135
63.0-100	0	0	0	0	0
>100	0	0	0	0	0
Micropore	49.02422039	39.68755835	26.08837424	23.64606792	20.55174115
Mesopore	39.60212877	45.12656288	34.36752121	30.30199534	25.93779322
Macropore	11.37365084	15.18587877	39.54410455	46.05193674	53.51046564

Table D S-3 Nuclear magnetic resonance test raw data
(Raw data of Figure 5(E) and Figure 5(F)

Free soaking time /month	Rock specimen number	Natural weight / g	Soaking weight / g	water absorption / %	Average water absorption / %
	S-1-1	544. 77	546. 98	0. 41	
1	S-1-2	544. 43	547. 18	0. 51	0. 49
	S-1-3	556. 19	559. 33	0. 56	
	S-3-1	555. 88	558. 78	0. 52	
3	S-3-2	549. 41	552. 45	0. 55	0. 57
	S-3-3	560. 38	563. 95	0. 64	
	S-6-1	543. 17	546. 94	0. 69	
6	S-6-2	545. 25	548. 45	0. 59	0. 63
	S-6-3	560. 49	563. 9	0. 61	
	S-9-1	542. 63	546.86	0. 78	
9	S-9-2	536. 86	541. 88	0. 94	0. 83
	S-9-3	540. 94	545. 06	0. 76	

Table E Water absorption rate of rock samples under different free immersion durations

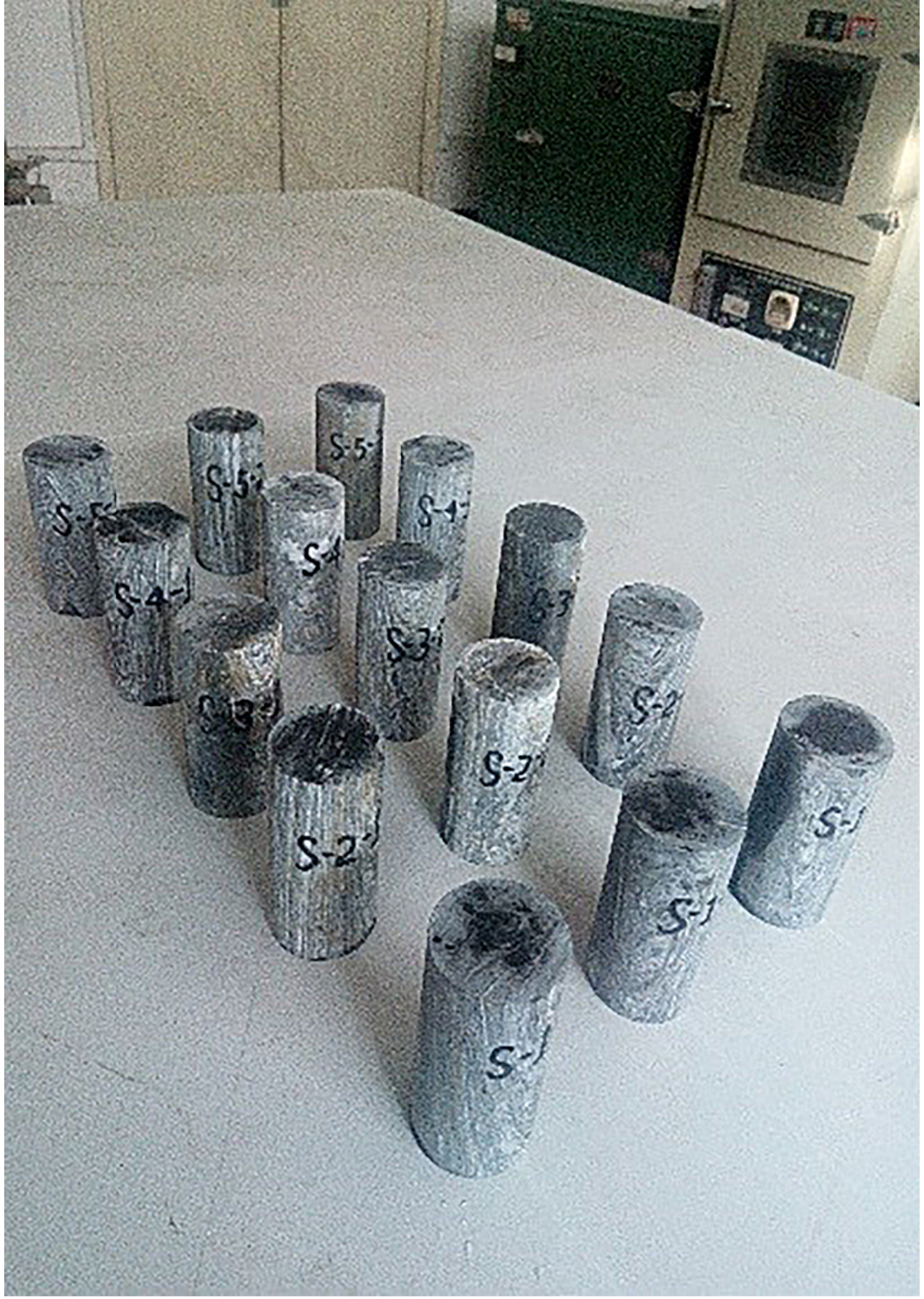

Figure F(A)   Cylindrical test block with the diameter of 50mm and height of 100mm
(Selected test block after numbering)

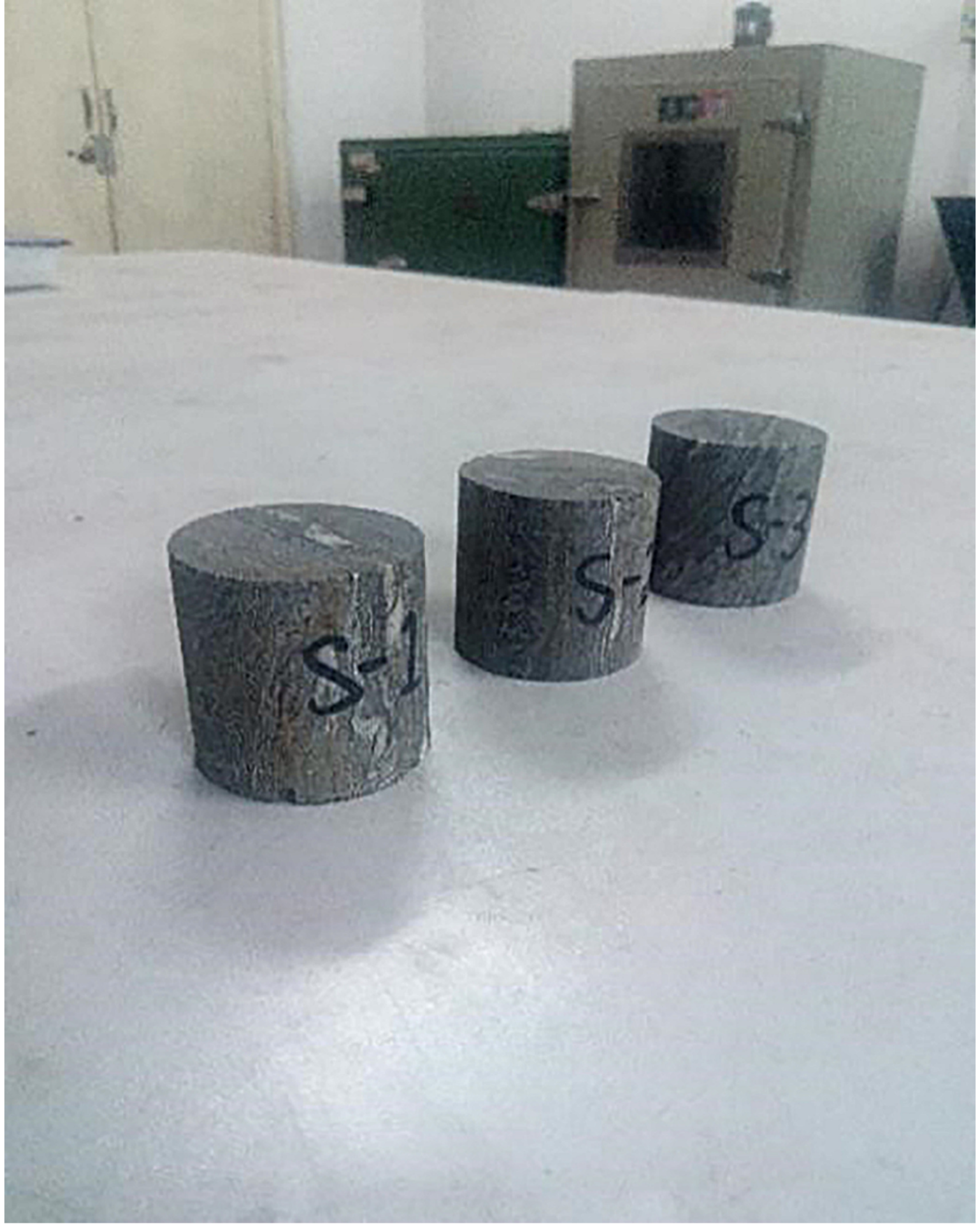

Figure F (B) Cylindrical test block with the diameter of 50mm and height of 50mm
(Selected test block after numbering)

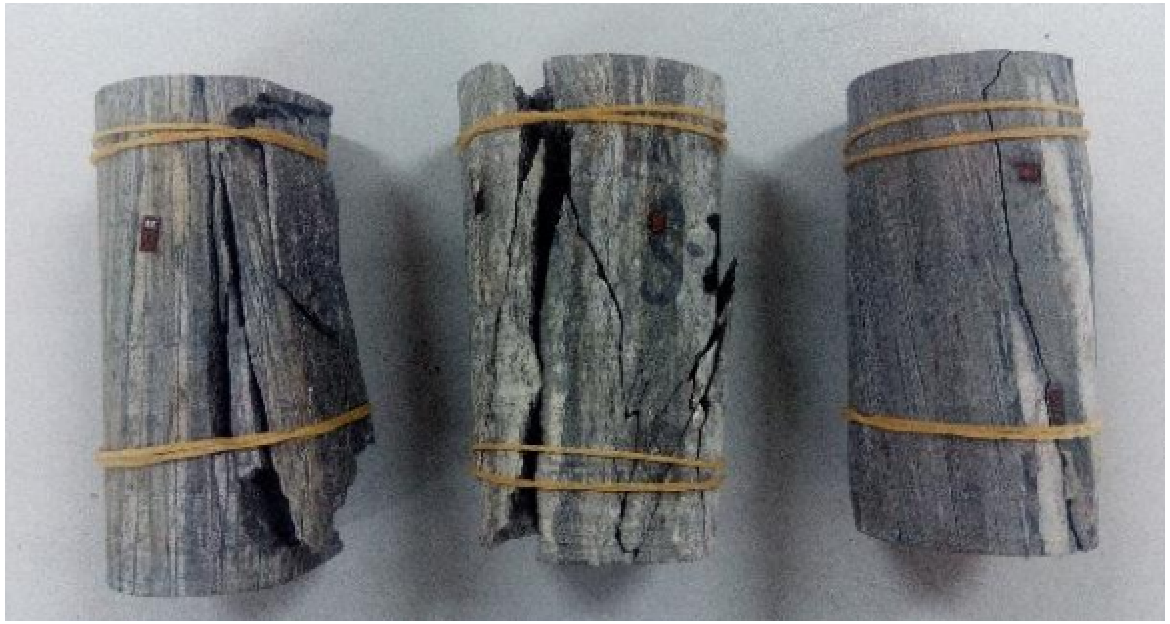

Figure G(a) The failure mode of the test block immersed in free soaking for 1 month

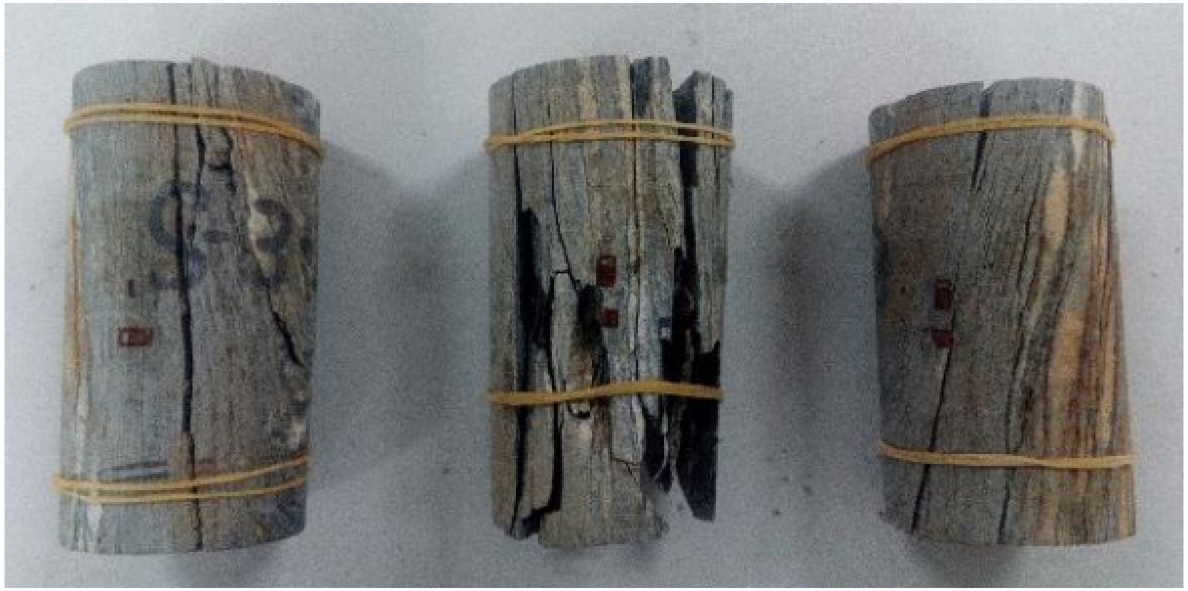

Figure G(b) The failure mode of the test block immersed in free soaking for 3 month

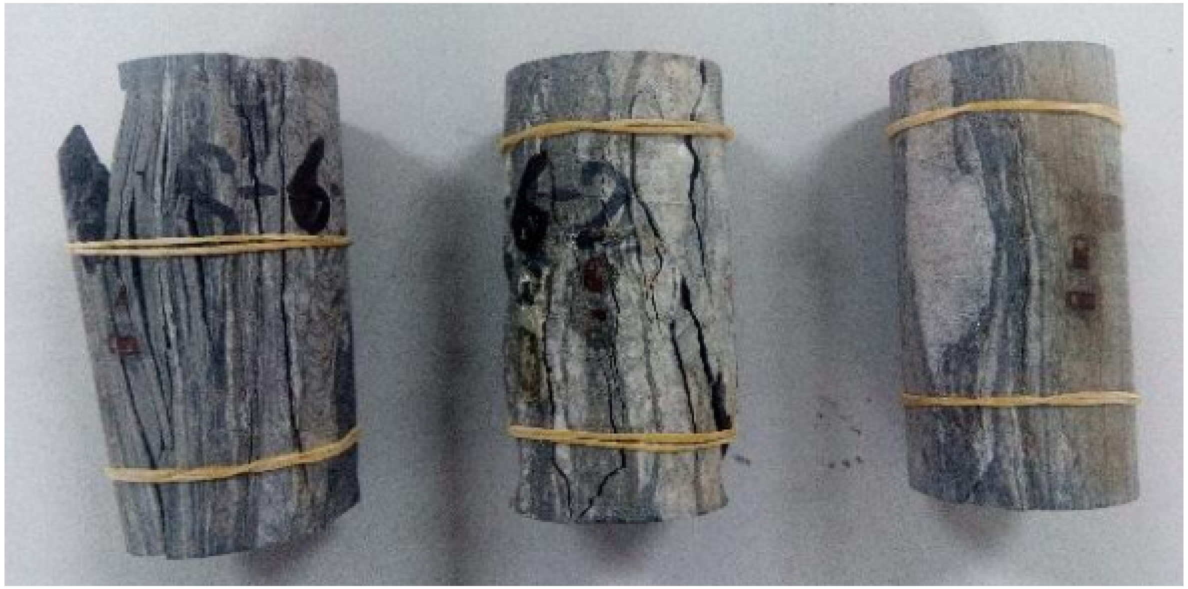

Figure G(c) The failure mode of the test block immersed in free soaking for 6 month

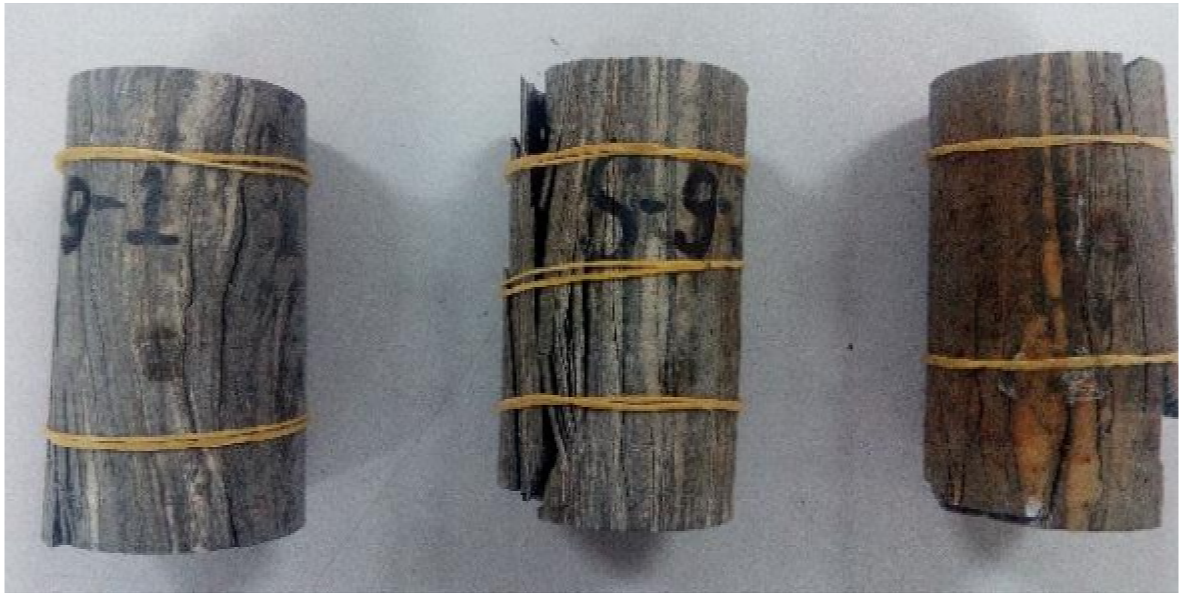

Figure G(d) The failure mode of the test block immersed in free soaking for 9 month
